# p16 Overexpression in Sinonasal Squamous Cell Carcinoma: Association with Human Papillomavirus and Prediction of Survival Outcomes

**DOI:** 10.3390/jcm12216861

**Published:** 2023-10-30

**Authors:** Hitoshi Hirakawa, Taro Ikegami, Masatomo Touyama, Yurika Ooshiro, Tomoyo Higa, Teruyuki Higa, Shinya Agena, Hidetoshi Kinjyo, Shunsuke Kondo, Norimoto Kise, Katsunori Tanaka, Hiroyuki Maeda, Tomoko Tamaki, Naoki Wada, Mikio Suzuki

**Affiliations:** 1 Department of Otorhinolaryngology, Head and Neck Surgery, Graduate School of Medicine, University of the Ryukyus, 207 Uehara, Nishihara-cho, Nakagami-gun, Okinawa 903-0215, Japan; aoi23@med.u-ryukyu.ac.jp (H.H.); ikegami@med.u-ryukyu.ac.jp (T.I.); puyoraer99110@gmail.com (M.T.); h067410@eve.u-ryukyu.ac.jp (T.H.);; 2 Department of Pathology and Oncology, Graduate School of Medicine, University of the Ryukyus, 207 Uehara, Nishihara-cho, Nakagami-gun, Okinawa 903-0215, Japanwadan@med.u-ryukyu.ac.jp (N.W.)

**Keywords:** sinonasal squamous cell carcinoma, human papillomavirus infection, p16 overexpression, nasal cavity, increasing trend, disease prognosis

## Abstract

p16 overexpression is often used as a surrogate marker for human papillomavirus (HPV) infection in oropharyngeal squamous cell carcinoma but remains an uncertain diagnostic tool for HPV-related sinonasal squamous cell carcinoma (SNSCC). Our study involved 79 consecutive SNSCC patients who were treated at a tertiary referral university hospital during 2006–2021. We retrospectively examined their clinical characteristics and conducted p16 immunohistochemistry and HPV detection. We found that 12.7% of the patients exhibited p16 overexpression, which was significantly more common in the nasal cavity and increased from 2015 onward. The HPV was a high-risk type and viral loads ranged from 4.2 to 1.6 × 10^6^ copies/ng DNA with genome integration. Five-year overall survival (OS) and five-year relapse-free survival (RFS) rates were 74.6% and 69.9%, respectively. Our multivariate analysis showed that T category (T1–4a) and hemoglobin levels (≥13.7) were significant favorable prognostic factors for OS, while T category, performance status, and p16 overexpression were significantly associated with RFS. In patients with p16 overexpression, OS was 100% and RFS was 90%. Our findings suggest that p16 overexpression is a reliable surrogate marker for transcriptionally active HPV infection and predicts a favorable prognosis.

## 1. Introduction

Sinonasal malignancies, although infrequent, account for about 3–5% of all head and neck cancers [1]. From 2004 to 2008, the annual incidence rate for sinonasal cancer was between 5 and 10 cases per million in males and 2 and 5 cases per million in females [2]. Sinonasal squamous cell carcinoma (SNSCC) is the predominant histological subtype, comprising 35–60% of all cases [3]. Notably, the incidence of sinonasal cancer exhibits marked regional variations, with the incidence of sinonasal malignancies appearing to be relatively high in the Asia–Pacific region compared with Europe and North America [2]. Radical surgery is the first choice of treatment, but alternative treatment approaches have been sought because of challenges such as aesthetic considerations, ethnic diversity, and the difficulty of securing clear surgical margins.

High-risk human papillomavirus (HPV) types are well-established culprits in oropharyngeal squamous cell carcinoma (OPSCC) [4]. In recent years, these high-risk HPV strains have been posited as potential drivers of SNSCC onset [3,5,6,7,8]. According to recent meta-analyses, the HPV prevalence in SNSCC cases is around 25.5% [5]. Moreover, HPV status seems to have a tangible correlation with survival outcomes, exhibiting modest heterogeneity across studies [8]. p16-positive malignant tumors in the larynx as well as OPSCC demonstrated an improved survival outcome after treatment [9]. Nevertheless, there is a wide range of reported HPV infection rates in SNSCC, owing to methodological differences and the limited samples in each study. Notably, the geographic variability in HPV prevalence seen in SNSCC mirrors the patterns seen in OPSCC [6], adding complexity in pinning down a direct association between high-risk HPV strains and SNSCC. In the diagnostic realm, p16 immunohistochemistry is favored for its cost-effectiveness and broad applicability, making it the go-to method for identifying transcriptionally active HPV infection in OPSCC [10]. However, p16 immunoreactivity is not a consistent marker for transcriptionally active high-risk HPV infection in sinonasal samples [11].

This study investigated in detail the diagnostic precision of p16 immunohistochemistry for detecting HPV DNA. Additionally, we aimed to shed light on the clinical features and survival outcomes associated with HPV-related SNSCC.

## 2. Materials and Methods

### 2.1. Study Design

**Figure 1:**
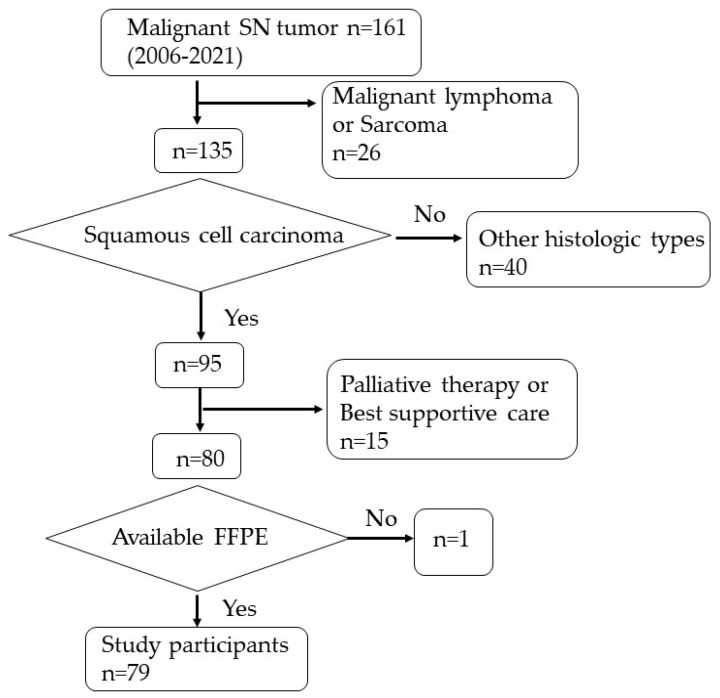
Selection of study participants. FFPE, formalin-fixed paraffin-embedded sample; *n*, number; SN, sinonasal.

In this retrospective observational study, we reviewed the clinical records of patients diagnosed with SNSCC (both keratinizing and nonkeratinizing SCC according to the update in the 5th edition of the WHO classification [12]) between August 2006 and November 2021 at a single tertiary referral university hospital (Ryukyu University Hospital). From this pool, 79 consecutive patients with SNSCC who underwent surgery or chemoradiotherapy (CRT) with curative intent were selected. We meticulously excluded patients with non-SCC tumor histology. Furthermore, we also excluded patients receiving palliative care due to distant metastasis or general complications and those who opted for the best supportive care (Figure 1). SNSCC consisted of 59% of all sinonasal malignant tumors.

All participants provided written informed consent before the collection and analysis of tumor samples. This study was approved by the Institutional Review Board of the University of the Ryukyus (project ID: 156) and complied with the ethical guidelines of the 1975 Declaration of Helsinki, as revised in 2008.

### 2.2. Clinical Evaluation

A multidisciplinary team, consisting of head and neck surgeons, radiation oncologists, and reconstructive surgeons, participated in pre-treatment evaluation and decision-making. This evaluation entailed an exhaustive review of the patient’s medical history, physical examination, performance status assessment, serum chemistry profiles, complete blood cell count, chest X-ray, computed tomography (CT), and magnetic resonance imaging (MRI). Positron emission tomography combined with CT was performed to identify lymph nodes or distant metastases.

Data on clinical parameters and oncological outcomes were recorded up to the end of the follow-up period on 31 July 2023. We determined clinical and pathological tumor staging in line with the Union for International Cancer Control (UICC) TNM Classification (eighth edition, 2017) [13].

Several parameters for nutritional status and general condition were assessed before treatment, including hemoglobin (Hb), albumin (Alb), neutrophil–lymphocyte ratio (NLR) [14], C-reactive protein (CRP), geriatric nutritional risk index (GNRI) [15], modified Glasgow prognostic score (mGPS) [16], prognostic nutritional index (PNI) [17] and Eastern Cooperative Oncology Group Performance Status (ECOG PS) [18,19]. PNI is derived from serum Alb levels and lymphocyte count [20], while NLR is the ratio of absolute neutrophil count to absolute lymphocyte count. These universally employed indices gauge inflammatory and nutritional status in cancer patients.

After primary treatment, we regularly checked for locoregional recurrence and distant metastasis through physical examinations, CT and/or MRI, and head and neck endoscopic examinations.

### 2.3. Surgical Treatment

Patients requiring total maxillectomy underwent combined neck dissection with reconstructive surgery in addition to removal of the primary lesion. Endoscopic sinus surgery was chosen for limited primary lesions where assured surgical margins were anticipated. Typically, postoperative radiation therapy (60 Gy), supplemented with platinum infusion (100 mg/m^2^, two or three times triweekly), was applied to the primary site and/or neck within 6 weeks postoperatively for cases with close/positive surgical margins or extracapsular lymph node extension. In patients with renal issues and those aged over 75 years, radiation therapy was prioritized as the postoperative adjuvant treatment instead of CRT.

### 2.4. CRT Protocol

This protocol, which has been described previously [21], is summarized here. The irradiation focus encompassed the maxilla, ethmoid sinus, nasal cavity, and pterygopalatine fossa, delivering 70 Gy across 35 sessions over 7 weeks. Along with the primary lesion, metastatic neck lesions were also treated. At the start of intensity-modulated radiotherapy, intra-arterial chemotherapy was introduced via a femoral route. Both docetaxel (60 mg/m^2^) and nedaplatin (80 mg/m^2^) were administered to the target lesions through the deployed microcatheters two or three times every 4 weeks.

### 2.5. p16 Immunohistochemistry

**Figure 2:**
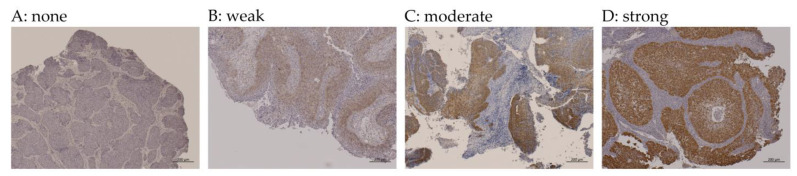
Representative cases in p16 immunohistochemistry. p16 immunoreactivity was classified according to positive cell percentage and staining intensity of the tumor nucleus and cytosol ((**A**), none; (**B**), weak; (**C**), moderate; (**D**), strong). p16 overexpression was defined as having more than 75% of tumor cells positive with moderate to strong staining as in the representative cases.

We assessed p16 expression using the CINtec^®^ p16 histology kit (Roche Applied Science, Penzberg, Germany) [11]. The threshold for p16 overexpression (both cytoplasmic and nuclear expression) was set as diffuse (≥75%) tumor expression with a minimum of moderate (+2/3) staining intensity, consistent with the 8th edition of the AJCC classification for OPSCC [13,22]. p16 immunoreactivity was classified according to the percentage of p16-positive cells out of all tumor cells and the intensity of p16 immunoreactivity (none, weak, moderate, or strong) (Figure 2).

### 2.6. HPV DNA Detection

Cases showing p16 overexpression underwent further testing by polymerase chain reaction (PCR) analysis and HPV DNA in situ hybridization. For PCR, DNA extracted from fresh frozen tumor samples was processed using the MY09/MY11 and GP5+/GP6+ degenerate consensus primer sets targeting the L1 region, as documented in previous studies [22]. The full experimental conditions are available in Supplementary Materials (Table S1).

We also introduced a novel quantitative real-time PCR assay system targeting the *E6* and *E2* genes of HPV strains 16, 18, 33, and 52. The assay system was designed to quantify the viral load and discern the viral integration status [11] (Table S2). For in situ hybridization analysis of high-risk HPV DNA, we employed the GenPoint HPV Biotinylated DNA Probe (Dako; Agilent Technologies, Inc., Santa Clara, CA, USA). This probe can identify an array of HPV types in formalin-fixed paraffin-embedded sections to confirm the presence of HPV DNA in SNSCC [11]. Details of the experimental conditions are presented in the Supplementary Materials.

In this research, HPV-related SNSCC was defined as cases exhibiting both p16 overexpression and the presence of HPV DNA as determined by PCR or in situ hybridization [11].

### 2.7. Statistical Analysis

Categorical data were analyzed using Pearson’s chi-squared test, and continuous data were analyzed using the Mann–Whitney U test. For comparisons with small sample sizes, Fisher’s exact test was used. Survival curves were plotted by the Kaplan–Meier method, and survival distributions were compared by the log-rank test. We used a multivariate Cox proportional hazard model to analyze prognostic factors and treatments influencing overall survival (OS) and relapse-free survival (RFS). Events were defined as death due to any cause for OS and as recurrence at the primary site, neck lymph node recurrence, or distant metastasis for RFS.

A *p-*value of <0.05 was considered statistically significant, and all analyses were performed using JMP Pro 15.0.0 (SAS Institute Inc., Cary, NC, USA).

## 3. Results

### 3.1. Patient Demographics, Clinical Testing and Treatment Profiles

The clinical characteristics of the patients are shown in Table 1. In our study cohort of seventy-nine participants, there were seventy men and nine women, with a median age of sixty-one (range: 30–90) years. Tumor locations were distributed as follows: maxillary sinus (sixty-three patients, 79.7%), ethmoid sinus (ten patients, 12.7%), and nasal cavity (six patients, 7.6%). The histologic classification was keratinizing squamous cell carcinoma in 75 patients (93.6%). There were no significant differences in tobacco and alcohol consumption, performance status, and histological differentiation between HPV-related and HPV-unrelated tumors. A notable finding was that p16 overexpression was significantly more prevalent in the nasal cavity (50.0%) than in the maxillary or ethmoid sinus (9.6%, *p* = 0.004, chi-squared test). A temporal trend was observed, with p16 overexpression becoming more common in patients treated after 2015 (*p* = 0.037, Fisher’s exact test).

Most patients were diagnosed with T4 (58.2%) and stage IV disease (68.4%). Treatment modalities included radiation-based treatment (RT/CRT) in 61 patients (77.2%), with the remaining 18 patients (22.8%) undergoing radical surgery followed by adjuvant RT/CRT-based treatment according to the pathological findings. Laboratory and nutritional variables did not show significant differences between the two tumor types (Table 2). There was no significant difference in inflammatory and nutritional status between HPV-related and HPV-unrelated tumors (Table 2).

### 3.2. p16 Overexpression and HPV DNA Detection

Of the 79 patients with SNSCC, 10 (12.7%) exhibited p16 overexpression. Figure 2 shows representative examples of p16 immunoreactivity. Table 3 shows the distribution of p16 immunoreactivity according to positive cell percentage and intensity. Some immunoreactivity to p16 was observed in 45.5% of the SNSCC cases, but these p16-positive cases usually had weak to moderate intensity and less than 50% positive cells (Table 3).

**Figure 3:**
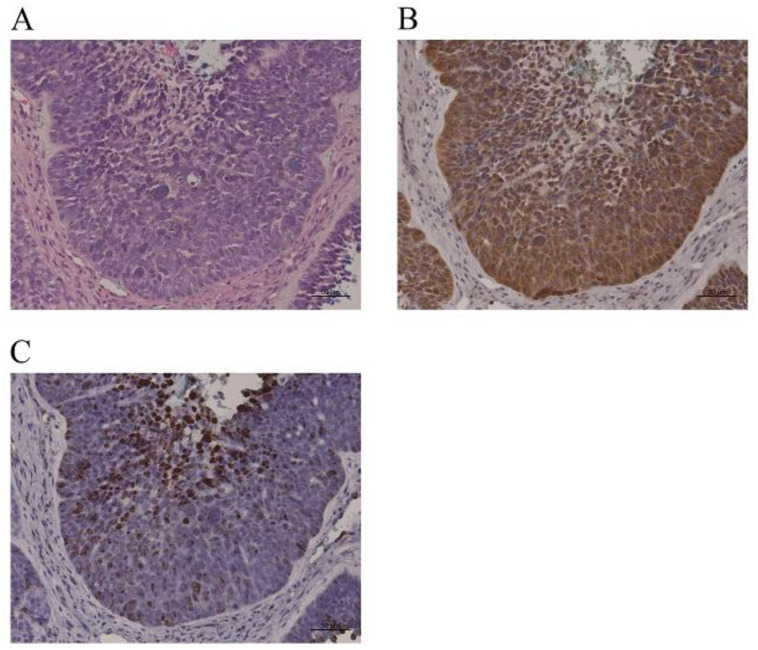
Representative findings for p16 overexpression in sinonasal squamous cell carcinoma. (**A**): hematoxylin–eosin staining. Bar = 50 µm. (**B**): p16 immunohistochemistry. Bar = 50 µm. (**C**): HPV DNA in situ hybridization. Bar = 50 µm. More than 75% of tumor cells expressed p16 immunoreactivity with moderate intensity and had a positive reaction in HPV DNA in situ hybridization.

Every p16-overexpressing sample tested positive for high-risk HPV DNA. The distribution of HPV types included HPV-16, HPV-18, HPV-33, and HPV-52, with some unidentified types. Viral loads across samples ranged from 4.2 to 1.63 × 10^6^ copies/ng genomic DNA, with each case displaying viral integration into the host genome (Table 4). Figure 3 shows representative images of p16 overexpression and HPV DNA in situ hybridization.

### 3.3. Prognostic Evaluation of Clinical Characteristics

**Figure 4:**
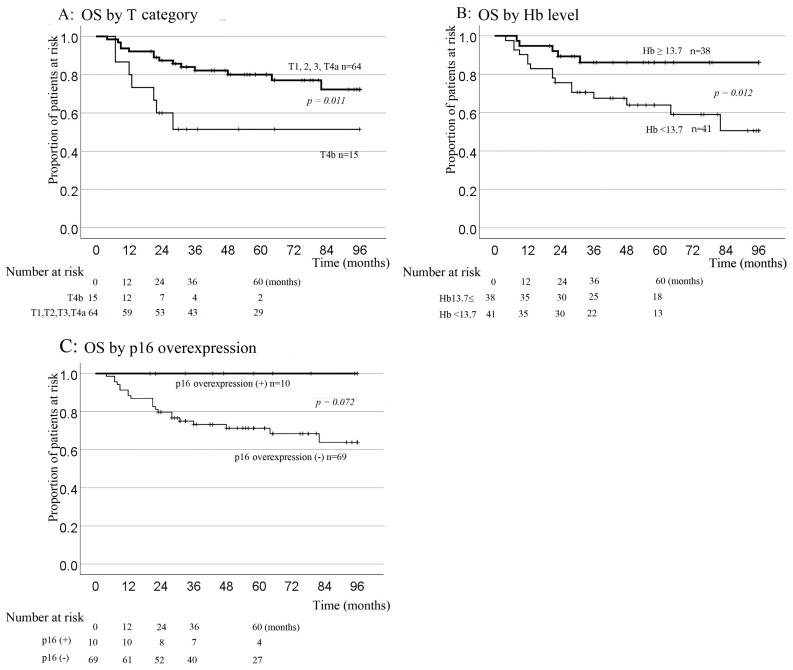
Kaplan–Meier cumulative survival plots for OS according to T category (**A**), Hb level (**B**), and p16 overexpression status (**C**). The 5-year OS tended to be better in the p16-positive group (100%) than in the p16-negative group (71.2%) (*p* = 0.072). Hb, hemoglobin; OS, overall survival.

Over a median follow-up duration of 60 months, the 5-year OS rate for the entire cohort was 74.6%. Univariate analysis identified T4b lesions and Hb levels below 13.7 g/dL as significant factors for poorer OS (*p* = 0.011 and *p* = 0.012, respectively). Although the 5-year OS rate for the p16-overexpression group was 100% (compared with 71.2% for the p16-negative group), this difference failed to achieve statistical significance by the log-rank test (*p* = 0.072, Figure 4).

In multivariate analysis considering the T category, Hb levels, and CRP, the significant prognostic factors for OS were T1–4a (*p* = 0.019) and Hb levels ≥ 13.7 g/dL (*p* = 0.035). Since all patients with p16 overexpression were alive, the multivariate analysis did not provide a hazard rate.

**Figure 5:**
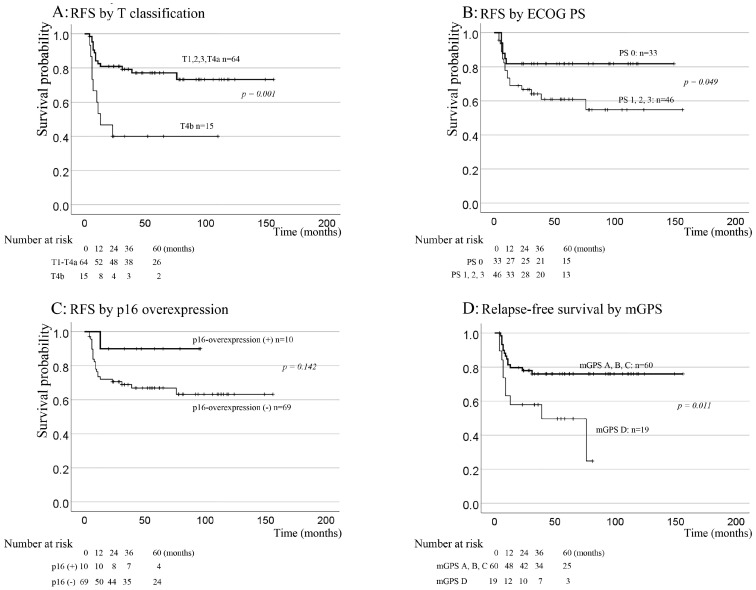
Kaplan–Meier cumulative survival plots for RFS according to the T category (**A**), ECOG PS (**B**), p16 overexpression (**C**), and mGPS (**D**). The five-year RFS rate was 69.9%. RFS in the p16-overexpression group was 90%, compared with 66.9% in the p16-negative group. However, the difference in RFS according to p16 overexpression was not significant in the univariate analysis (*p* = 0.142) but was significant in the multivariate analysis (*p* = 0.012). mGPS, modified Glasgow prognostic score; ECOG PS, Eastern Cooperative Oncology Group Performance Status; RFS, relapse-free survival.

The 5-year RFS rate was 69.9%. In univariate analysis, the T category, ECOG PS, Hb levels, Alb levels, and mGPS displayed significant prognostic implications, as shown in Figure 5 and Table 5.

As visualized in Figure 5, the Kaplan-Meier curves revealed a 5-year RFS of 90% for the p16-overexpression group, compared with 66.9% for the p16-negative group. However, p16 overexpression was not significant in the univariate analysis (*p* = 0.142). Since these nutritional indicators confound each other, mGPS and ECOG PS were included in the multivariate analysis. In the multivariate analysis of RFS, which included the T category, Hb levels, mGPS, ECOG PS, and p16 overexpression, T1–4a (*p* = 0.004), ECOG PS 0 (*p* = 0.026), and p16 overexpression (*p* = 0.012) were identified as significant prognostic markers.

## 4. Discussion

The reported prevalence of HPV in SNSCC varies widely, ranging from 8.9% to 62% [5,23,24,25,26,27,28]. This considerable discrepancy can be attributed to differing definitions of HPV-related SNSCC, methodological differences in HPV detection, and small sample sizes. When studies focus solely on *E6/E7* mRNA expression of the HPV gene, which is considered to most accurately reflect active transcriptional infection, prevalence rates tend to decrease by approximately 25% [7,25,29]. While the RNA-based test is recognized as the gold standard for accuracy, its complexity and cost prohibit its widespread use in the clinical setting [7]. Our study underscores the strong association of p16 overexpression with the presence of high-risk HPV DNA, suggesting that p16 immunohistochemistry might be a cost-effective, sensitive method for pinpointing transcriptionally active HPV infections in SNSCC. In the present study, p16 overexpression was present in 12.7% of SNSCC cases. Considering the small number of p16overexpressing cases and the cost-effectiveness of detecting HPV-related SNSCC, p16 immunohistochemistry should be examined first, and if p16 overexpression is found, PCR for HPV DNA or HPV DNA in situ hybridization tests are recommended. However, the number of patients with p16 overexpression was low (*n* = 10), and thus there might be a risk for bias in the present results. Further study is needed to confirm the importance of p16 overexpression in SNSCC, as shown in the present study, including the survival benefit, the characteristics of tumor location, and the increasing trend.

In OPSCC, the role of p16 overexpression as a surrogate marker for detecting active HPV infection has been well-established due to its robust predictive and prognostic value [4]. However, its precise cut-off value for overexpression in SNSCC has not yet been defined, which may account for the variance in p16 positivity rates among different studies. This lack of clarity might lead to reduced sensitivity of p16 immunohistochemistry as an indicator of transcriptionally active HPV in SNSCC as opposed to OPSCC. In the present study, approximately half of the cases had p16 immunoexpression, and moderate intensity of p16 expression was also observed in HPV-unrelated cases (Table 3). However, when the threshold for p16 overexpression was set at diffuse (≥75%) tumor expression with a minimum of moderate (+2/3) staining intensity, all samples with p16 overexpression had high-risk HPV strains with viral integration into the genome by PCR or DNA in situ hybridization, and these cases had a favorable prognosis. These results aligned with RNA-based tests, affirming their potential for effective survival prognosis stratification. In the present study, T4b and nutritional status were significantly associated with survival outcomes, which is consistent with previous reports [30,31]. Previous reports have found that low Hb and high CRP levels were associated with poor prognosis in head and neck cancer [32,33]. The results of the present study are consistent with those reports. A low Hb level has been linked to malnutrition, weight loss, and cancer cachexia [34], and a high CRP level reflects systemic inflammation. These nutritional and inflammatory markers might be important in the prognosis of SNSCC. In addition to these factors, p16 overexpression was an independent prognostic factor for RFS in multivariate analysis and linked to superior OS, as all p16-positive SNSCC of our cohort are alive. The literature has shown a possible survival advantage linked to p16 expression [24,27,28,30]. However, several reports have shown no survival benefits of p16 expression [23,35]. These discrepant findings could be attributed to differences in patient demographics, HPV testing methodologies, or classifications across studies. Our findings highlight the survival advantages of p16 overexpression in SNSCC, echoing the benefits seen in HPV-related OPSCC cases [4,22]. In the future, the definition of p16 overexpression in SNSCC should be clarified in the same way as in OPSCC through studies with larger cohorts and a unified detection protocol.

Insights into the viral load and integration status of SNSCC remain scarce. In this investigation, we provided data on HPV DNA viral load and viral integration status for SNSCC with p16 overexpression (Table 4). Our observed HPV types align with the high-risk subtypes detected in OPSCC at our institution [22]. Drawing parallels from our prior study on oropharyngeal carcinoma, the HPV-16 viral loads in OPSCC varied between 0.3 × 10^5^ and 2.69 × 10^5^ copies/ng DNA (median 2.666 × 10^4^ copies/ng DNA). In contrast, the present study’s HPV-16 and HPV-33 viral loads in SNSCC ranged from 4.2 to 1.63 × 10^6^ copies/ng DNA. Notwithstanding the limited sample size, viral loads in SNSCC did not significantly deviate from those in OPSCC, and all types showing p16 overexpression exhibited viral integration. These insights affirm the critical role of HPV, particularly in its relation to p16 overexpression, in shaping the landscape of SNSCC.

The location of HPV-related sinonasal cancer remains unclear. Previous reports showed no significant difference in HPV prevalence among sinonasal regions [7]. Recently, the prevalence of HPV was found to be higher in the nasal cavity than in the maxillary sinus by analysis of the National Cancer Database with various HPV detection methods [28]. According to a recent report from Italy [36], only 3% of nasal septum tumor was classified as SCC. In the present study, high-risk HPV was most frequently detected in the nasal cavity, which is in line with a recent meta-analysis, and this is the first report of this finding in Asia. Given the geographical and racial discrepancies in sinonasal carcinoma and HPV infection, further study is needed to clarify these aspects.

The availability of HPV status in OPSCC increased with each subsequent year of diagnosis from 2008: 0.1–0.2% for 2004–2008, 1.4% for 2009, 30.6% for 2010, 45.6% for 2011, and 56.9% for 2012 [37]. Recently, an increase in the incidence of HPV-associated SNSCC and the prevalence of HPV-positive SNSCC from 1995 to 2019 has been demonstrated using the SEER (Surveillance, Epidemiology, and End Results Program) database in the United States [38]. The results of the present study on the prevalence of HPV-related SNSCC in an Asian country are consistent with those in the United States. This might reflect the widespread HPV infection in the head and neck. We must pay attention to HPV infection in the entire head and neck region, not only the oropharynx.

## 5. Conclusions

p16 overexpression combined with HPV DNA testing might be a reliable surrogate marker for HPV-related SNSCC and an accurate prognostic marker. High-risk HPV infection was the most frequently detected in the nasal cavity, and the prevalence of HPV-related SNSCC is increasing in Japan as in the United States. A larger multicenter prospective study is needed to clarify the prognostic significance of high-risk HPV infection in SNSCC patients treated with a multimodal approach.

## Figures and Tables

**Table 1:** Clinical profiles in sinonasal squamous cell carcinoma patients.

Variables	Total SNSCC (*n* = 79)	HPV-Related Tumor (*n* = 10)	HPV-Unrelated Tumor (*n* = 69)	*p-*Value
Sex				
Male	70	7	63	0.048
Female	9	3	6	
Median age				0.525
≤61	39	4	35	
>61	40	6	34	
Smoking history				
Yes	64	6	58	0.070
No	15	4	11	
Alcohol consumption				
Yes	67	8	59	0.650
No	12	2	10	
ECOG PS				
0	33	4	29	0.903
1, 2, 3	46	6	40	
Primary subsite				
Maxillary sinus	63	6	57	
Ethmoid sinus	10	1	9	
Nasal cavity	6	3	3	
Maxillary and ethmoid sinus	73	6	66	0.004
Nasal cavity	6	3	3	
Clinical T category				
T1	3	1	2	
T2	8	2	6	
T3	22	4	18	
T4a	31	1	30	
T4b	15	2	13	
T1, T2, T3, T4a	64	8	56	0.930
T4b	15	2	13	
Clinical N category				
N0	52	6	46	
N1	11	2	9	
N2	15	2	13	
N3	1	0	1	
N0	52	6	46	0.678
N1–3	27	4	23	
Clinical UICC stage				
I	3	1	2	
II	9	2	7	
III	13	2	11	
IVA, IVB	54	5	49	
I, II, III	23	5	18	0.120
IV	56	5	51	
Histology				
Keratinizing	75	8	67	* 0.076
Non-keratinizing	4	2	2	
Treatment				0.822
CRT/RT	61	8	53	
Surgery ± RT/CRT	18	2	16	
First medical examination				* 0.037
Before 2015	35	1	34	
2015 or later	44	9	35	
Prognosis				
Alive without disease	58	10	48	
Died of disease	19	0	19	
Died of other cause	2	0	2	

CRT, chemoradiotherapy; ECOG PS, Eastern Cooperative Oncology Group Performance Status; RT, radiation therapy; SNSCC, sinonasal squamous cell carcinoma; UICC, 8th edition of the Union for International Cancer Control; * Fisher’s exact test; other categorical data were analyzed by chi-squared tests.

**Table 2:** Laboratory and nutritional variables in SNSCC.

Variables	Total SNSCC (*n* = 79)	HPV-Related Tumor (*n* = 10)	HPV-Unrelated Tumor (*n* = 69)	*p-*Value
Hb (g/dL)				
Median	13.7			
<13.7	41	6	35	0.583
≥13.7	38	4	34	
NLR (%)				
Median	3.19			
<3.2	40	5	35	0.966
≥3.2	39	5	34	
Alb (g/dL)				
Median	3.9			
<3.9	37	4	33	0.643
≥3.9	42	6	36	
CRP (mg/dL)				
Median	1.2			
≤0.78	37	6	31	0.372
≥0.78	42	4	38	
GNRI				
<98	31	3	28	0.522
≥98	48	7	41	
mGPS				
A, B, C	60	6	54	0.207
D	19	4	15	
PNI				
<40	8	2	6	0.268
≥40	71	8	63	

Alb, albumin; CRP, C-reactive protein; GNRI, geriatric nutritional risk index; Hb, hemoglobin; mGPS, modified Glasgow prognostic score, NLR, neutrophil–lymphocyte rate; PNI, prognostic nutritional index; SNSCC, sinonasal squamous cell carcinoma.

**Table 3:** Distribution of p16 immunoreactivity.

	Intensity Score	None	Weak	Moderate	Strong	Total
% of positive cells	0	43	0	0	0	43
	1–20	0	12	6	0	18
	20–50	0	3	5	0	8
	50–75	0	0	0	0	0
	≥75	0	0	5	5	10
	Total	43	15	16	5	79

**Table 4:** HPV analysis in p16 overexpression cases.

Case	Sex	Age	First Visit (Year)	Primary Site	HPV Type	Viral Load (Copies/ng DNA)	*E2/E6*	ISH
1	M	64	2014	Nasal cavity	HPV-52	61,694.74	0.747	Positive
2	M	61	2015	MS	NA	NA	NA	Positive
3	M	66	2016	MS	NA	NA	NA	Positive
4	F	46	2016	ES	HPV-18	2627.9	0	Positive
5	M	65	2018	Nasal cavity	HPV-16	141.6	0.0511	Positive
6	F	63	2019	MS	NA	NA	NA	Positive
7	F	53	2019	Nasal cavity	HPV-33	1.63 × 10^6^	0.8307	Positive
8	M	80	2020	MS	NA	NA	NA	Positive
9	M	46	2021	MS	HPV-16	4.3	0	Positive
10	M	64	2021	MS	HPV-16	4.2	0	Positive

ES, ethmoid sinus; F, female; ISH, in situ hybridization; M, male; MS, maxillary sinus; NA, not available.

**Table 5:** Univariate and multivariate analyses for OS and RFS.

	OS			RFS		
	Univariate Analysis	Multivariate Analysis		Univariate Analysis	Multivariate Analysis	
Factor (Category)	*p-*Value	*p-*Value	HR (95% CI)	*p*-Value	*p*-Value	HR (95% CI)
Sex (male vs. female)	0.335			0.547		
Age (≤61 vs. ≥62 years)	0.848			0.702		
Tobacco history (yes vs. no)	0.529			0.333		
Alcohol consumption (yes vs. no)	0.479			0.663		
ECOG PS (0 vs. 1, 2, 3)	0.273			0.049	0.026	0.364 (0.140–0.945)
Primary subsite (nasal cavity vs. other)	0.652			0.516		
Histology (keratinizing vs. non-keratinizing)	0.921			0.815		
p16 overexpression or not	0.072	0.004	NA	0.142	0.012	0.137 (0.018–1.073)
T category (T1, T2, T3, T4a vs. T4b)	0.011	0.019	3.365 (1.312–8.630)	0.001	0.004	4.016 (1.68–9.61)
N category (N0 vs. N, 1, 2, 3)	0.736			0.862		
UICC clinical stage (I, II, III vs. IV)	0.067			0.087		
Hb (<13.7 vs. ≥13.7 g/dL)	0.012	0.035	3.129 (1.016–9.6370)	0.008	0.074	2.458 (0.891–6.777)
NLR (≤3.2% vs. ≥3.2%)	0.101			0.069		
Alb (<3.9 vs. ≥3.9 g/dL)	0.104			0.020		
CRP (≥0.78 vs. <0.78 mg/dL)	0.058	0.615	0.765 (0.265–2.210)	0.095		
GNRI (<98 vs. ≥98)	0.357			0.379		
mGPS (A, B, C vs. D)	0.166			0.011	0.187	0.540 (0.217–1.341)
PNI (<40 vs. ≥40)	0.26			0.013		
Treatment modality (RT/CRT vs. surgery + RT/CRT)	0.575			0.836		

Alb, albumin; CRP, C-reactive protein; CRT, chemoradiotherapy; ECOG PS, Eastern Cooperative Oncology Group Performance Status; GNRI, geriatric nutritional risk index; Hb, hemoglobin; HR, hazard ratio; mGPS, modified Glasgow prognostic score; NA, not available; NLR, neutrophil–lymphocyte rate; OS, overall survival; PNI, prognostic nutritional index; RFS, relapse-free survival; RT, radiation therapy; SNSCC, sinonasal squamous cell carcinoma; UICC, 8th edition of the Union for International Cancer Control.

## Data Availability

The datasets generated and/or analyzed during the present study have not been made publicly available. However, data can be made available from the corresponding author upon reasonable request.

## Supplementary Materials

The following supporting information can be downloaded at https://www.mdpi.com/article/10.3390/jcm12216861/s1, Supplementary Methods; Table S1: Primers used for the detection of HPV DNA by PCR; Table S2: Primers used for the detection of viral loads and viral integration in real-time PCR.
