# Supplementary material for: p16 Overexpression in Sinonasal Squamous Cell Carcinoma: Association with Human Papillomavirus and Prediction of Survival Outcomes
Source: J Clin Med. 2023 Oct 30;12(21):6861. doi: 10.3390/jcm12216861 (PMC10650892; doi:10.3390/jcm12216861)
Supplement: Supplementary file 1 [file jcm-12-06861-s001.zip › jcm-2663546-supplementary.pdf]

Journal of
Clinical Medicine

MDPI

Supplementary Materials

p16 Overexpression in Sinonasal Squamous Cell Carcinoma: Association with Human Papillomavirus and Prediction of Survival Outcomes

Hitoshi Hirakawa [1,†], Taro Ikegami [1,†], Masatomo Touyama [1], Yurika Ooshiro [1], Tomoyo Higa [1], Teruyuki Higa [1], Shinya Agena [1], Hidetoshi Kinjyo [1], Shunsuke Kondo [1], Norimoto Kise [1], Katsunori Tanaka [1], Hiroyuki Maeda [1], Tomoko Tamaki [2], Naoki Wada [2] and Mikio Suzuki [1,*]

[1] Department of Otorhinolaryngology, Head and Neck Surgery, Graduate School of Medicine, University of the Ryukyus, 207 Uehara, Nishihara-cho, Nakagami-gun, Okinawa 903-0215, Japan; aoi23@med.u-ryukyu.ac.jp (H.H.); ikegami@med.u-ryukyu.ac.jp (T.I.); puyoraer99110@gmail.com (M.T.); h067410@eve.u-ryukyu.ac.jp (T.H.)

[2] Department of Pathology and Oncology, Graduate School of Medicine, University of the Ryukyus, 207 Uehara, Nishihara-cho, Nakagami-gun, Okinawa 903-0215, Japan; wadan@med.u-ryukyu.ac.jp (N.W.)

* Correspondence: suzuki@med.u-ryukyu.ac.jp; Tel.: +81-895-1183

† These authors contributed equally to this work.

Contents

Supplementary Methods

Table S1. Primers used for the detection of HPV DNA by PCR.

Table S2. Primers used for the detection of viral load and viral integration in real-time PCR.

Supplementary Methods for HPV Infection Analysis

Polymerase Chain Reaction for HPV DNA Detection

Samples in patients with p16 overexpression were then analyzed by polymerase chain reaction (PCR) using fresh frozen samples. DNA was extracted from the fresh frozen samples using a Gentra Puregene Tissue Kit (Qiagen, Germantown, MD), and the presence and viral integration of DNA were examined in all samples. The DNA extraction areas were required to contain at least 30% tumor nuclei out of all cell nuclei. If there were insufficient fresh frozen samples for PCR, HPV DNA in-situ hybridization and PCR were carried out using formalin-fixed, paraffin-embedded (FFPE) sections and DNA extracted from FFPE samples, respectively.

FFPE samples were macrodissected from 10-µm sections, and DNA was extracted using the GeneRead DNA FFPE Kit (Qiagen).

The presence of DNA in all samples was assessed by PCR *β-globin* gene amplification using the primers PC04 and GH20 (Table S1) [1]. Negative (water) and positive (DNA of HPV-16-positive CaSki cell line) controls were included in each amplification series. The CaSki cell line was purchased from the European Collection of Cell Cultures (Salisbury, Wiltshire, UK). The cell line has been tested and authenticated by DNA (STR) profiling at the JCRB Cell Bank (Osaka, Japan).

The general consensus primer sets GP5+/GP6+ and MY09/11 were used to detect the presence of HPV DNA by PCR as described previously (Table S1) [1]. In addition, negative DNA samples in the GP5+/GP6+ or MY09/MY11 PCR were re-amplified in a nested PCR using the GP5+/GP6+ primer pair. PCR products of the expected size (GP5+/GP6+, 150 bp; MY09/MY11, 450 bp) were purified and directly sequenced with an ABI PRISM 3130xl Genetic Analyzer (Applied Biosystems, Foster City, CA). The sequences were then

aligned, and they were compared with those of known HPV types in the GenBank database using the BLAST program.

*Determination of Viral Load and Integration *

Viral load DNA was measured, and the physical status of HPV-16-infected samples was clarified as described previously [1]. Briefly, quantitative real-time PCR assays were established for the *E6* and *E2* genes of HPV-16, HPV-18, HPV-33 and HPV-52. Quantitative real-time PCR was performed with the CFX96 Touch™ Real-Time PCR Detection System (Bio-Rad, Hercules, CA). The PCR reaction mixture (10 µL) contained 0.2 µM primers, 5.0 µL SYBR Premix Ex Taq™ II (Tli RNaseH plus; Takara, Otsu, Japan) and 2.0 µL standard plasmid DNA or genomic DNA (30 ng). The PCR profile was as follows: 95 °C for 30 s followed by 40 cycles at 95 °C for 5 s and 60 °C for 30 s. Specific amplification of each HPV DNA was verified by melting curve analysis and gel electrophoresis of the products. Details of primer information, detection range and amplification efficiency are shown in Table S2. An external standard curve was created using serial dilutions (0.3, 3, 30 and 300 ng) of human genomic placental DNA (Sigma-Aldrich, St. Louis, MO; Merck KGaA, Darmstadt, Germany) for cellular DNA quantification, and *β-globin* was amplified as an internal control. Viral DNA load was assessed by calculating the *E6* copy number [1]. The total *E6* copy number in 1 ng cellular DNA was determined. Then, we calculated the ratio of the *E2* copy number to the total *E6* copy number. An *E2*/*E6* ratio ≥ 1 denotes predominance of the episomal form, whereas 0 < *E2*/*E6* ratio < 1 demonstrates a mix of both integrated and episomal forms. A ratio of 0 indicates the presence of the integrated form only.

In Situ Hybridization with HPV DNA Probes

Biotinyl-tyramide-based in situ hybridization was performed using GenPoint™ HPV biotinylated DNA probe and GenPoint™ tyramide signal amplification system for biotinylated probes according to manufacturer's instruction (Dako Japan, Tokyo, Japan). The GenPoint™ HPV biotinylated DNA probe reacted HPV types 16, 18, 31, 33, 35, 39, 45, 51, 52, 56, 58, 59 and 68 in FFPE sections by in situ hybridization as described previously [1]. Serial 4-µm-thick sections of FFPE samples were deparaffinized in xylene and a graded alcohol series. Target HPV DNA retrieval was performed in 10 mM sodium citrate (at pH 6.0) at 95 °C for 40 min. The slides were digested with Proteinase K (10,000 times dilution with Tris-buffered saline, Dako Japan) for 10 min at room temperature. Endogenous peroxidases were blocked with 0.3% H_2O_2 in methanol for 20 min. A drop of the HPV probe was added to the section and a coverslip was applied. The probe and the target DNA were denatured by incubating the slides at 92 °C for 5 min. After denaturation, the slides were transferred to a humidified chamber for hybridization at 37 °C for 16 h. After hybridization, coverslips were removed and slides were then bathed in Tris-buffered saline containing 0.05% Tween-20 (TBST). Then, a stringent wash was performed using GenPoint™ Detection System stringent wash solution (Dako Japan) at 48 °C for 30 min followed by rinsing in TBST. Detection of the hybridized probe was then performed using the GenPoint™ Detection System according to the kit instructions, using primary streptavidin-horseradish peroxidase (HRP), biotinyl-tyramide, secondary streptavidin-HRP and DAB (3,3-diaminobenzidine; Dako Japan). Slides were counterstained with hematoxylin.

Immunohistochemistry for $p16^{INK4a}$

Immunohistochemistry for $p16^{INK4a}$ was performed using CINTec® p16 Histology kit (MTM Laboratories AG, Heidelberg, Germany). Serial 4-µm-thick sections of FFPE samples were deparaffinized in xylene and a graded alcohol series, followed by heating at 95–99 °C for 10 min in epitope retrieval solution as described previously [1]. Endogenous peroxidases were blocked with peroxidase blocking reagents. The sections were incubated for 30 min at room temperature with primary monoclonal mouse anti-$p16^{INK4a}$ antibody.

Following washes in phosphate-buffered saline, the slides were incubated at room temperature for 30 min with an HRP-conjugated goat anti-mouse secondary antibody (MTM Laboratories AG, Heidelberg, Germany). Immunolabeling was visualized by incubation in DAB for 10 min. Stained slides were counterstained with hematoxylin.

Table S1. Primers used for the detection of HPV DNA by PCR.

Primers	Sequence
GP5+	5′-TTTGTTACTGTGGTAGATACTAC-3′
GP6+	5′-GAAAAATAAACTGTAAATCATATTC-3′
MY09	5′-CGTCCMARRGGAWACTGATC-3′
MY11	5′-GCMCAGGGWCATAAYAATGG-3′
PC04	5′-CAACTTCATCCACGTTCACC-3′
GH20	5′-GAAGAGCCAAGGACAGGTAC-3′

Table S2. Primers used for the detection of viral load and viral integration in real-time PCR.

Real-time PCR primers	Sequence
HPV-16 *E6*-qPCR-F	5′-GAGAACTGCAATGTTTCAGGACC-3′
HPV-16 *E6*-qPCR-R	5′-TGTATAGTTGTTTGCAGCTCTGTGC-3′
HPV-16 *E2*-qPCR-F	5′-AACGAAGTATCCTCTCCTGAAATTATTAG-3′
HPV-16 *E2*-qPCR-R	5′-CCAAGGCGACGGCTTTG-3′
HPV-18 *E6*-qPCR-F	5′-TGAAAAACGACGATTTCACAAC-3′
HPV-18 *E6*-qPCR-R	5′-TACTTGTGTTTCTCTGCGTCGT-3′
HPV-18 *E2*-qPCR-F	5′-GCAGCTACACCTACAGGCAAC-3′
HPV-18 *E2*-qPCR-R	5′-CGCTATGTTTTCGCAATCTGT-3′
HPV-33 *E6*-qPCR-F	5′-AAACCACGAACATTGCATGA-3′
HPV-33 *E6*-qPCR-R	5′-TCCAAATGGATTTCCCTCTCT-3′
HPV-33 *E2*-qPCR-F	5′-TGGAAACTGATACGCATGGA-3′
HPV-33 *E2*-qPCR-R	5′-GCAATGTCCATTGGCTTGTA-3′
HPV-52 *E6*-qPCR-F	5′-TCAAACGCCATTATGTCCTG-3′
HPV-52 *E6*-qPCR-F	5′-GGGGTCTCCAACACTCTGAA-3′
HPV-52 *E2*-qPCR-F	5′-AGAAACGACGACGACCAGAC-3′
HPV-52 *E2*-qPCR-R	5′-TCAGTTGCAGTGACGAGTCC-3′

References

1. Hirakawa, H.; Ikegami, T.; Kise, N.; Kinjyo, H.; Kondo, S.; Agena, S.; Hasegawa, N.; Kawakami, J.; Maeda, H.; Suzuki, M. Human papillomavirus infection and EGFR exon 20 insertions in sinonasal inverted papilloma and squamous cell carcinoma. *J Pers Med* **2023**, *13*, 65
